# Supplementary material for: Characterization and Comparative Genomics Analysis of a New Bacteriophage BUCT610 against Klebsiella pneumoniae and Efficacy Assessment in Galleria mellonella Larvae
Source: Int J Mol Sci. 2022 Jul 21;23(14):8040. doi: 10.3390/ijms23148040 (PMC9321532; doi:10.3390/ijms23148040)
Supplement: Supplementary file 1 [file ijms-23-08040-s001.zip › ijms-1792708-supplementary.pdf]

Table S1. The results of lifestyle identification of phage BUCT610.

Sample Name	Target Name	Reporter	CT	Sample Name	Target Name	Reporter	CT	Sample Name	Target Name	Reporter	CT
BUCT610	terminase sunbit	SYBR	11.235	BUCT610	conserved hypothetical protein	SYBR	9.485	BUCT610	recombinase	SYBR	7.447
BUCT610	terminase sunbit	SYBR	11.144	BUCT610	conserved hypothetical protein	SYBR	9.418	BUCT610	recombinase	SYBR	7.365
BUCT610	terminase sunbit	SYBR	11.174	BUCT610	conserved hypothetical protein	SYBR	9.513	BUCT610	recombinase	SYBR	7.445
K1119	terminase sunbit	SYBR	37.187	K1119	conserved hypothetical protein	SYBR	37.376	K1119	recombinase	SYBR	38.048
K1119	terminase sunbit	SYBR	37.081	K1119	conserved hypothetical protein	SYBR	37.525	K1119	recombinase	SYBR	Undetermined
K1119	terminase sunbit	SYBR	37.281	K1119	conserved hypothetical protein	SYBR	37.505	K1119	recombinase	SYBR	Undetermined
R-K1119-RA4	terminase sunbit	SYBR	Undetermined	R-K1119-RA4	conserved hypothetical protein	SYBR	35.406	R-K1119-RA4	recombinase	SYBR	35.262
R-K1119-RA4	terminase sunbit	SYBR	Undetermined	R-K1119-RA4	conserved hypothetical protein	SYBR	35.424	R-K1119-RA4	recombinase	SYBR	36.565
R-K1119-RA4	terminase sunbit	SYBR	Undetermined	R-K1119-RA4	conserved hypothetical protein	SYBR	35.624	R-K1119-RA4	recombinase	SYBR	36.505
R-K1119-RA9	terminase sunbit	SYBR	Undetermined	R-K1119-RA9	conserved hypothetical protein	SYBR	37.518	R-K1119-RA9	recombinase	SYBR	34.459
R-K1119-RA9	terminase sunbit	SYBR	39.906	R-K1119-RA9	conserved hypothetical protein	SYBR	37.696	R-K1119-RA9	recombinase	SYBR	34.395
R-K1119-RA9	terminase sunbit	SYBR	39.606	R-K1119-RA9	conserved hypothetical protein	SYBR	37.780	R-K1119-RA9	recombinase	SYBR	34.383
R-K1119-RB5	terminase sunbit	SYBR	Undetermined	R-K1119-RB5	conserved hypothetical protein	SYBR	Undetermined	R-K1119-RB5	recombinase	SYBR	Undetermined
R-K1119-RB5	terminase sunbit	SYBR	Undetermined	R-K1119-RB5	conserved hypothetical protein	SYBR	37.454	R-K1119-RB5	recombinase	SYBR	36.155
R-K1119-RB5	terminase sunbit	SYBR	Undetermined	R-K1119-RB5	conserved hypothetical protein	SYBR	37.435	R-K1119-RB5	recombinase	SYBR	36.055
R-K1119-RB7	terminase sunbit	SYBR	Undetermined	R-K1119-RB7	conserved hypothetical protein	SYBR	35.711	R-K1119-RB7	recombinase	SYBR	35.515
R-K1119-RB7	terminase sunbit	SYBR	Undetermined	R-K1119-RB7	conserved hypothetical protein	SYBR	35.665	R-K1119-RB7	recombinase	SYBR	35.521
R-K1119-RB7	terminase sunbit	SYBR	Undetermined	R-K1119-RB7	conserved hypothetical protein	SYBR	35.727	R-K1119-RB7	recombinase	SYBR	35.519

The results of qPCR to determine the lifestyle of phage BUCT610. The experiment was repeated three times for each sample. (samples: R-K1119-RA4; R-K1119-RA9; R-K1119-RB5; R-K1119-RB7; Negative group: K1119; Positive group: BUCT610).
